# Molecular epidemiology of *Babesia microti* in southern Zhejiang: an integrated survey of humans, rodent reservoirs, and tick vectors (2020–2023)

**DOI:** 10.3389/fmicb.2026.1799424

**Published:** 2026-03-20

**Authors:** Jia-Qi Zhang, Xuan Zhang, Wen-Jie Xu, Hua-Liang Chen, Yan Feng, Ke-Gen Yu, Qiao-Yi Lu, Ji-Min Sun, Xiao-Xiao Wang, Wei Ruan

**Affiliations:** Zhejiang Provincial Center for Disease Control and Prevention, Hangzhou, Zhejiang, China

**Keywords:** apicomplexa, asymptomatic infections, *Babesia microti*, babesiosis, epidemiology, genetic diversity, tick-borne pathogens

## Abstract

**Background:**

*Babesia*, an intraerythrocytic protozoan, is globally distributed and poses a significant public health burden. In China, human babesiosis is mainly caused by *Babesia microti*, and the Zhejiang Province is endemic. This study aimed to determine the prevalence of *B. microti* in the population, reservoir hosts, and vectors; assess population exposure risks; and gain further insight into the genotype structure in Zhejiang.

**Methods:**

From May 2020 to October 2023, blood samples from local residents and blood donors, as well as blood or liver tissues from rodents and questing or host-attached ticks, were collected from seven counties in southern Zhejiang Province. DNA was extracted for the detection of *B. microti* and identification of the rodent and tick species. A retrospective investigation was conducted on individuals who tested positive. Positive samples were sequenced for phylogenetic analyses.

**Results:**

In total, 4,728 samples were collected, including 2,475 local residents, 358 blood donors, 1,615 rodents, and 280 questing or host-attached ticks. *B. microti* was detected in six local residents, 71 rodents, and nine ticks, with infection rates of 0.24, 4.40, and 3.21%, respectively. Human infections and positive ticks were found in specific counties, whereas positive rodents were detected in all counties. All six infected individuals were asymptomatic carriers with a history of outdoor activities in forested areas, and four also reported contact with animals. A total of 17 rodent species were identified, among which *Niviventer lotipes* exhibited the highest risk. The tick species identified included *Haemaphysalis longicornis*, *Ixodes granulatus*, *Rhipicephalus haemaphysaloides*, and *Rhipicephalus microplus*. Positive samples were detected for both *I. granulatus* and *R. microplus*. Sequencing and phylogenetic analyses of the positive samples revealed that all the sequences belonged to the Kobe type.

**Conclusion:**

This study is the first comprehensive report of *B. microti* infection across its life cycle in Zhejiang Province, revealing a wide prevalence in rodents and ticks. Asymptomatic infections in humans have also been reported. *N. lotipes* appears to be the predominant rodent reservoir host. In addition, *I. granulatus* and *R. microplus* may serve as primary tick vectors. These findings provide valuable information for developing preventive strategies against babesiosis in China.

## 1 Introduction

*Babesia* is an intraerythrocytic protozoan first identified in cattle and sheep erythrocytes in 1888. As apicomplexan parasites, *Babesia* spp. are mainly transmitted through tick bites and can potentially infect all vertebrate mammals, including humans (Hildebrandt et al., 2007). Globally, there are more than 100 species of *Babesia*; however, only a few species (*Babesia microti*, *Babesia duncani*, *Babesia divergens*, *Babesia venatorum* and the KO1 strain) have been implicated in human infections and are known to cause babesiosis (Vannier et al., 2015, Vannier and Krause, 2012, Homer et al., 2000). In addition to tick bites, babesiosis can be transmitted through blood transfusions, transplantation of organs from infected donors, or congenital (mother-to-child) transmission (Vannier et al., 2015). *Babesia* infection can be asymptomatic or can cause mild-to-severe illness that can be fatal (Vannier et al., 2015).

In recent years, the number of reported human babesiosis cases has increased annually, and babesiosis is considered a significant threat to public health worldwide (Chen et al., 2019). Cases of human babesiosis have been reported throughout the world (Vannier and Krause, 2012). Its incidence has increased exponentially in the United States over the past five decades (Joseph et al., 2011, 2020), and more cases have been reported in Europe (Hildebrandt et al., 2007, Häselbarth et al., 2007). Babesiosis has also been reported in significant numbers in Japan, Korea, and China (Wei et al., 2001, Hussain et al., 2021, Zhou et al., 2014). In China, *B. microti*, *B. venatorum*, and *B. divergens* are thought to be the main pathogens causing human babesiosis, *B. microti* appears to be the dominant pathogen in the Zhejiang Province (Chen et al., 2019). Since the first case of *B. microti* infection was reported in Zhejiang Province in 2011 (Yao et al., 2012), more cases of *B. microti* infection have been documented (Zhang et al., 2020), including fatal cases (Xu et al., 2023), most of which have occurred in the mountainous areas of southern Zhejiang.

The mountainous region of southern Zhejiang has been recognized as a major biodiversity hotspot. Dominated by mid- to low-altitude terrain (average elevation 500–1,200 m) and >75% forest cover, it features a warm, humid climate and complex ecosystems that constitute ideal habitats for tick vectors and rodent hosts (Wang et al., 2023). This area is not only the principal focus of tick-borne diseases in Zhejiang Province but also maintains a complete zoonotic transmission chain. In addition, local agriculture centered on tea and mushroom cultivation keeps residents in prolonged contact with the vector habitats. Collectively, these distinctive natural and anthropogenic factors make the mountains of southern Zhejiang an endemic area for *B. microti*.

As definitive hosts for *B. microti*, ticks are the primary vectors responsible for transmitting *B. microti* to vertebrates. Small rodents are the most critical reservoir hosts of *B. microti* (Vannier et al., 2015). The epidemiology of *B. microti* infections in Zhejiang remains largely unknown. The objective of this study was to determine the prevalence of *B. microti* in the population, reservoir hosts, and vectors; assess the population exposure risks; and gain further insight into the *B. microti* genotype structure in Zhejiang.

## 2 Materials and methods

### 2.1 Study area

**Figure 1:**
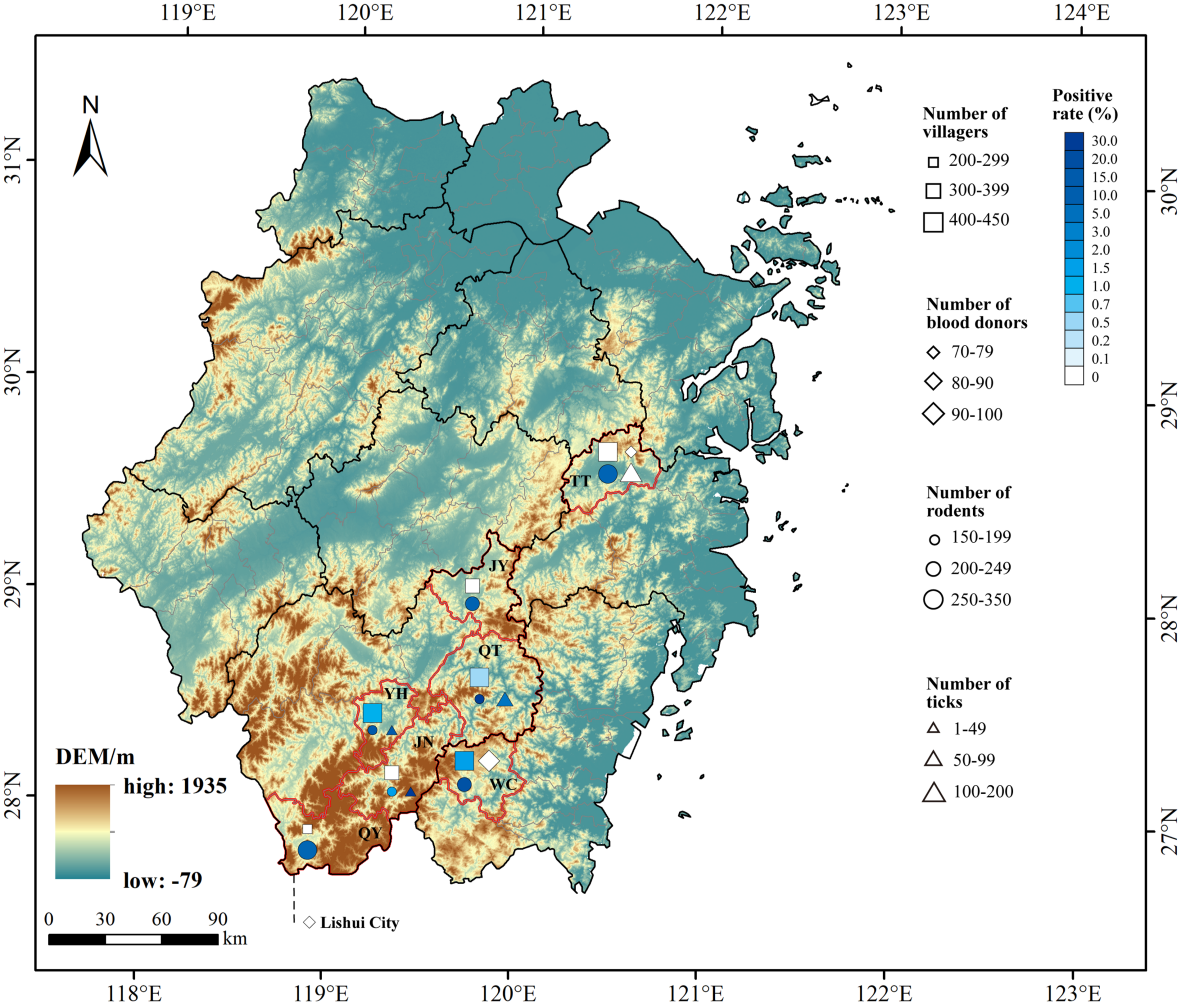
Geographic map of sampling sites with sample counts and *B. microti* positivity rates in the mountains of southern Zhejiang Province, China. □, ◇, ○, and △ represent the villagers, blood donors, rodents, and ticks, respectively. Colors indicate the positive rate. TT, Tiantai County; WC, Wencheng County; QT, Qingtian County; YH, Yunhe County; JY, Jinyun County; JN, Jingning County; QY, Qingyuan County.

Our study has focused on the southern mountainous region of the Zhejiang Province. Seven counties were enrolled based on the positive detection of *B. microti* in local reservoirs/vectors and documented reports of human babesiosis cases (Ruan et al., 2017), including Tiantai County in Taizhou; Wencheng County in Wenzhou; and Qingtian, Yunhe, Jinyun, Jingning, and Qingyuan counties in Lishui (Figure 1). One to three mountainous towns in each county were selected as sampling sites.

### 2.2 Sample collection

Blood samples from local residents, blood donors, rodent blood or liver tissues, and questing or host-attached ticks were collected from seven counties. Sampling was conducted annually between 2020 and 2023 during the seasonal activity period from May to October. Blood samples were obtained from local residents during annual physical examinations and blood donor samples were collected during routine pre-donation screening at local blood stations. A retrospective investigation was conducted on the individuals who tested positive for *B. microti*, focusing on symptoms, history of outdoor activities in forested areas, animal contact, tick bites, and any history of blood transfusion or organ transplantation (Supplementary file 1). The rodent samples were collected using traps in mountainous regions. Questing ticks sampling was performed using a white crib flannel sweep measuring 50 × 100 cm with a PVC pipe handle (Egizi et al., 2019), and host-attached ticks were collected from rodent samples by fine point forceps (Occhibove et al., 2022). Blood samples were collected in EDTA-K_2_ tubes, while liver tissue and ticks were stored in cryopreservation tubes at −20 °C until further use.

### 2.3 DNA extraction

Each tick was placed in 180 μL of Qiagen buffer ATL with 20 μL Qiagen Proteinase K (10 mg/mL) in microfuge tubes and homogenized with a TissueLyser (TIANGEN® TGrinder OSE-Y50, CHINA). DNA from individual blood, tissue, and ticks were extracted using Dneasy® Blood and Tissue kits (Qiagen, Germany), according to the manufacturer’s instructions. DNA was eluted from each column twice with 100 μL of Qiagen’s elution buffer AE into separate labeled microtubes at −20 °C until further use.

### 2.4 Species identification of rodents and ticks

Rodent DNA was amplified using the primer pair 16SarL/16SHm in order to generate partial mitochondrial 16S rRNA sequences (549 bp) for species identification (Quérouil et al., 2001). Tick DNA was amplified using the primer pair 16 + 1/16S-1, yielding partial mitochondrial 16S rRNA sequences (460 bp) for species identification (Black and Piesman, 1994). All amplicons were sequenced, and species identity was confirmed by BLAST analysis against the GenBank database (https://blast.ncbi.nlm.nih.gov/Blast.cgi).

### 2.5 Detection of *Babesia microti*

All DNA extracts were screened for *B. microti* using TaqMan-based real-time PCR (qPCR) assays with self-designed primers and probes targeting an 85 bp partial region of 18S rRNA. The real-time PCR was conducted in a QuantStudio™ 5 detection system (TermoFisher Scientific, USA). Samples were prepared in 20 μL reactions, which included 10 μL of 2 × Universal Probe qPCR Master Mix (Accurate Biology, AG11704, CHINA), 0.4 μL of 20 pmol of each oligonucleotide primer, 0.2 μL of 20 pmol of the fluorescence-labeled probe, 2 μL genomic DNA template, and 7 μL of RNase/DNase-free water. Temperature cycling parameters were 95 °C for 5 min, followed by 40 cycles of 95 °C for 10 s and 51 °C for 35 s.

The sensitivity and specificity of the qPCR assay were validated prior to application. Analytical sensitivity was first determined using 10-fold serial dilutions of a plasmid containing the target fragment, and the limit of detection was established at 10 copies/μL, corresponding to a Ct value slightly above 36. To further assess assay performance under clinical conditions, validation was also performed using blood samples from confirmed *Babesia microti* patients. Parasite density was estimated by microscopic counting, using 8,000 leukocytes per μL as the reference for parasitemia calculation. After serial dilution of these blood samples, the qPCR detection limit was observed at approximately Ct ≈ 36, corresponding to an estimated parasitemia of about 1–10 parasites/μL. To ensure reproducibility and minimize false-positive results near the detection threshold, a Ct value of 35 was conservatively defined as the diagnostic cut-off; samples with Ct ≤ 35 were considered positive. To evaluate assay specificity, cross-reactivity tests were performed using DNA from several other blood-borne protozoan parasites, including *Plasmodium* spp., *Toxoplasma gondii*, *Theileria* spp., and *Leishmania* spp. No cross-amplification was observed in these tests.

### 2.6 Sequencing and phylogenetic analysis of the *Babesia microti* based on 18S rRNA gene

For *B. microti* qPCR-positive samples amplified using the primer pair Prio2F/Prio6R-ref, nearly full-length (1700 bp) 18S rRNA gene sequences were obtained. The amplicons were sequenced using two additional primers: Ba-SL and Ba-SR (Wei et al., 2020).

Sequencing was conducted by Hangzhou Youkang Biological Co., LTD, and sequence assembly was performed using BioEdit 7.2.5 (USA). All primers and a probe were produced by TSINGKE Biological Co., LTD and are listed in Supplementary file 2.

Using GenBank’s Nucleotide search “*Babesia microti* & 18S” and “*Babesia microti* & small subunit ribosomal RNA” search term, a sequence-length filter ranging from 1,500 to 1800 bp was applied to retain sufficient informative sites for the phylogenetic analysis. A phylogenetic tree was constructed using the neighbor-joining method and the Kimura 2-parameter model in MEGA 11 (Pennsylvania State University, State College, PA, USA). The sequences obtained in this study were submitted to GenBank (accession numbers PQ039648-PQ039675).

### 2.7 Statistical analysis

Statistical analyses were performed using GraphPad Prism software (version 8.0; GraphPad Software, Inc., San Diego, CA, USA). Pearson’s chi-square test or Fisher’s exact test was used to examine the association between parasitic infections. *p* < 0.05 was considered statistically significant.

## 3 Results

### 3.1 General information

In total, 4,728 samples were collected from seven counties in Zhejiang Province from 2020 to 2023, including 2,475 from local residents, 358 from blood donors, 1,615 from rodents, and 280 from questing or host-attached ticks (Table 1; Figure 1).

**Table 1:** Prevalence of *B. microti* collected from the mountains of southern Zhejiang Province, 2020–2023.

Year	Villagers n/N (%)	Blood donors n/N (%)	Rodents n/N (%)	Ticks n/N (%)
2020	6/557 (1.08)	0/130 (0)	27/358 (7.54)	8/67 (11.94)
2021	0/502 (0)	0/49 (0)	15/392 (3.83)	0/16 (0)
2022	0/708 (0)	0	24/459 (5.23)	1/29 (3.45)
2023	0/708 (0)	0/179 (0)	5/406 (1.23)	0/168 (0)
Total	6/2475 (0.24)	0/358 (0)	71/1615 (4.40)	9/280 (3.21)

n/N (%): n tested positive/N total (%).

### 3.2 Demographics of local residents and blood donors

This study included 2,475 local residents with a mean age of 61.20 years and 358 blood donors with a mean age of 33.96 years. Among the local residents, 1,052 were men and 1,204 were women, yielding a male-to-female ratio of 1:1.14. The majority of residents participating in this survey had an education level of primary school or lower (57.9%), and the most common occupation was farmer (67.6%). In the blood donor group, 109 were men and 70 were women, yielding a male-to-female ratio of 1:1.56. Most had an education level of high school or higher (20.7%), and the main occupations were farmers and students (36.9%) (Table 2).

**Table 2:** Demographic of local residents and blood donors.

Variable	Local residents *N* = 2,475	Blood donors *N* = 358
Age		
Mean ± SD	61.20 ± 18.56	33.96 ± 11.86
Median [IQR]	66 [54, 73]	37 [20, 44]
Gender		
Male	1,052	109
Female	1,204	70
Unknown*	219	179
Education		
Illiterate	661	0
Primary school	773	5
Junior high school	284	37
Senior high school	150	19
College and above	9	18
Unknown*	598	279
Occupation		
Worker	104	11
Farmer	1,674	82
Businessperson	15	9
Forestry worker	1	0
Medical worker	0	19
Service worker	5	7
Homemaker	4	0
Retiree	3	0
Student	186	50
Preschool child	20	0
Other	2	0
Unknown*	461	180

*Due to missing responses from participants, some demographic data were incomplete and were recorded as “Unknown”.

### 3.3 Prevalence of *Babesia microti* in participants

**Figure 2:**
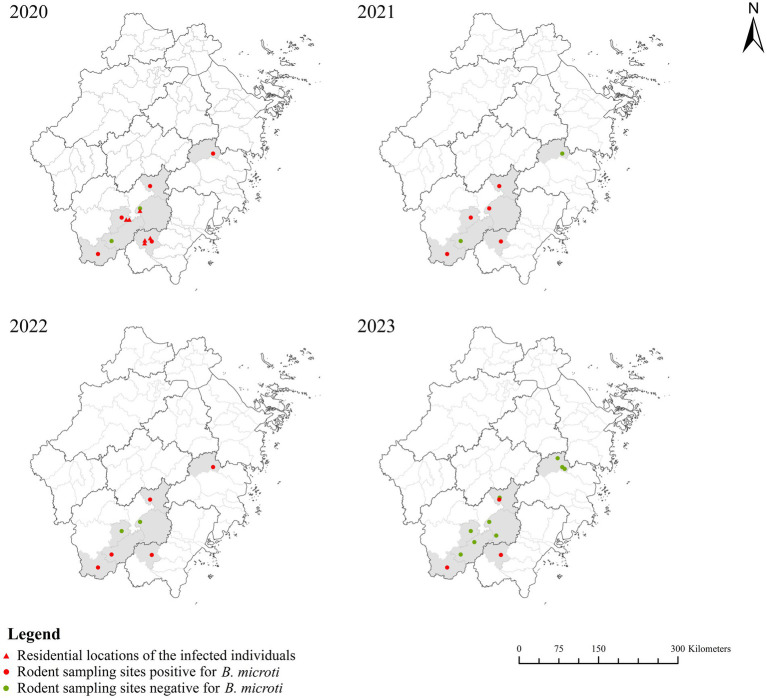
The locations of rodent sampling sites and infected individuals from 2020 to 2023.

Among the 2,475 local residents, six *B. microti* infections were detected, yielding a positivity rate of 0.24%. In contrast, none of the 358 blood donors tested positive for *B. microti* infection (Table 1). However, given the relatively limited sample size of blood donors, this finding should be interpreted with caution. Human infections were identified in Wencheng, Qingtian, and Yunhe counties (Figure 1), and the residential locations of the infected individuals are shown in Figure 2. Each of the six infected individuals was an asymptomatic carrier aged 63 years or older; additionally, five were illiterate and worked as farmers. All individuals had a history of outdoor activities in forested areas, and four had contact with animals, such as rodents, cats, dogs, or livestock. Two individuals denied any history of tick bites, and the remaining four individuals were uncertain about possible tick exposure. All six denied any history of blood transfusion or organ transplantation (Table 3).

**Table 3:** Demographic of six human infections.

Monitoring year	City	County	Gender	Age	Occupation	Education level	Symptoms	Activities in forested areas	Contact with animals	Tick* bites	Blood transfusion or organ transplantation
2020	Lishui	Yunhe	Female	63	Farmer	Illiterate	Asymptomatic	Yes	Rodents, cats, and dogs	Unknown	No
2020	Lishui	Yunhe	Male	63	Worker	Junior high school	Asymptomatic	Yes	Rodents, cats, and dogs	Unknown	No
2020	Lishui	Qingtian	Male	74	Farmer	Illiterate	Asymptomatic	Yes	No	No	No
2020	Wenzhou	Wencheng	Male	81	Farmer	Illiterate	Asymptomatic	Yes	No	No	No
2020	Wenzhou	Wencheng	Female	67	Farmer	Illiterate	Asymptomatic	Yes	Rodents and livestock	Unknown	No
2020	Wenzhou	Wencheng	Male	68	Farmer	Illiterate	Asymptomatic	Yes	Rodents, cats, and dogs	Unknown	No

*Unknown: tick bite history could not be determined; No: the individual explicitly denied having any history of tick bites.

### 3.4 Prevalence of *Babesia microti* in rodents

Among the 1,615 rodent samples, 71 were positive for *B. microti*—with positive detection in each sampled county—yielding an overall prevalence of 4.40% (Table 1). Infection rates varied across the four survey years, with a peak observed in 2020 (27/358, 7.54%). A similar temporal pattern was observed in villagers, with the highest infection rate also recorded in 2020 (6/557, 1.08%). Notably, 2020 was the only year in which *B. microti* infection was detected among villagers. The prevalence of *B. microti* varies among Tiantai, Qingtian, Yunhe, and Jingning, where positive rodents are not detected annually. In contrast, infected rodents were found in Wencheng, Jinyun, and Qingyuan during all four survey years (Figure 2). Wencheng County exhibited the highest *B. microti* infection prevalence among sampled rodents (9.48%, 22/232), with a pronounced peak in 2020 (15.49%, 11/71). Tiantai, Jinyun, Yunhe, and Qingyuan also showed relatively high infection rates—5.86, 5.37, 3.23 and 3.07%, respectively.

**Figure 3:**
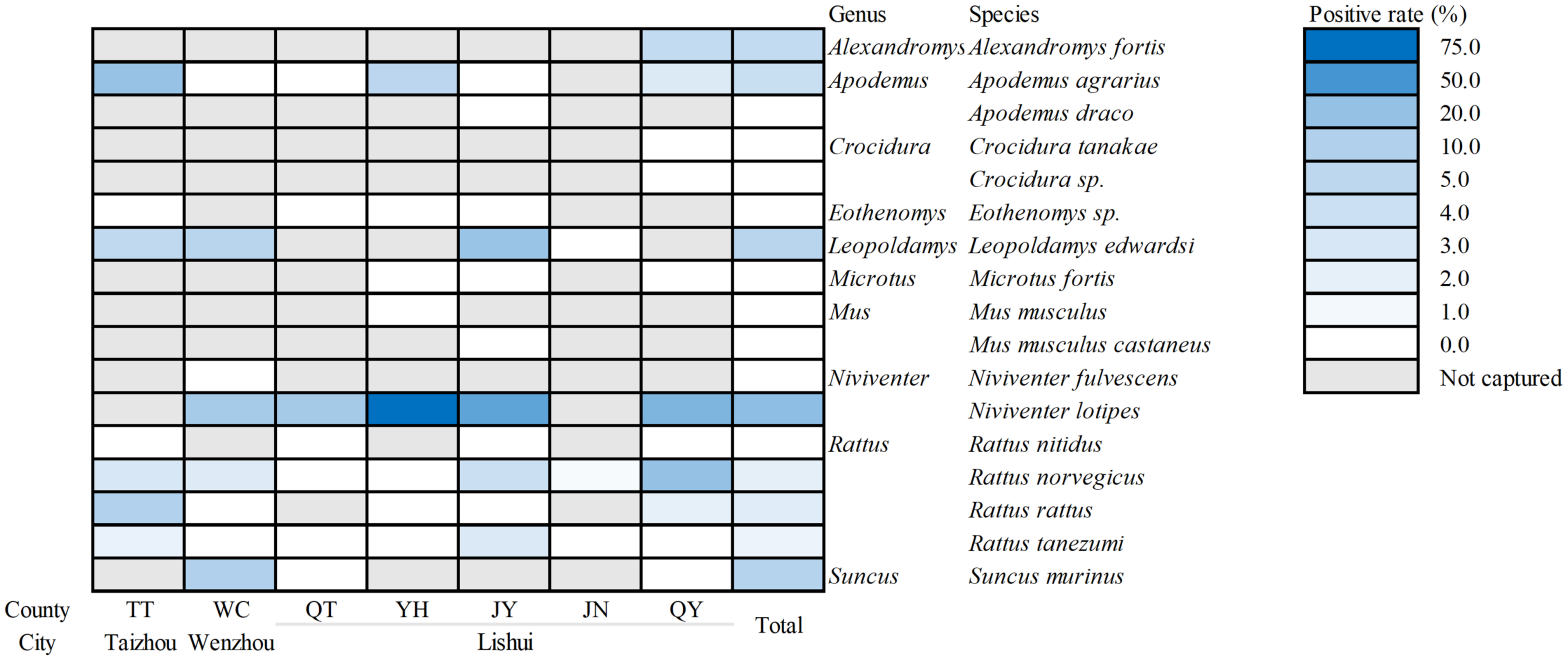
Prevalence of *B. microti* among rodent species across seven counties. The color indicates the positive rate, and the numbers indicate the sample sizes. TT, Tiantai County; WC, Wencheng County; QT, Qingtian County; YH, Yunhe County; JY, Jinyun County; JN, Jingning County; QY, Qingyuan County.

Due to the incomplete preservation of the DNA from 2020 and amplification or sequencing failures in some samples, 1,248 rodents were identified. These trapped rodents belonged to 17 species: the *Rattus norvegicus* accounted for the largest proportion of all species tested, comprising 34.78% (n = 434), whereas *Apodemus draco*, *Crocidura* sp. and *Mus musculus* made up the smallest proportion, each representing only 0.08% (*n* = 1). 56 rodents from 8 species, *Alexandromys fortis* (4.55%, 2/44), *Apodemus agrarius* (4.21%, 11/261), *Leopoldamys edwardsi* (7.41%, 6/81), *Niviventer lotipes* (22.45%, 22/98), *Rattus norvegicus* (2.07%, 9/434), *Rattus rattus* (2.41%, 2/83), *Rattus tanezumi* (1.50%, 2/133), *Suncus murinus* (8.00%, 2/25), were actively infected with *B. microti*. The infection rate of *N. lotipes* was significantly higher than that of the other rodent species. Among the four *Rattus* species trapped, *R. nitidus* was the only species for which no *B. microti*–positive samples were detected. Notably, two positive samples were also identified for *S. murinus*, despite the limited number of individuals trapped in this species (Table 4; Figure 3).

**Table 4:** Prevalence of *B. microti* in rodents of different species.

Genus	Species	No. of tested	No. of positive (%)	OR (95% CI)
*Alexandromys*	*Alexandromys fortis*	44	2 (4.55)	3.02 (0.46–19.59)
*Apodemus*	*Apodemus agrarius*	262	11 (4.20)	2.79 (0.67–12.76)
	*Apodemus draco*	1	0 (0)	
*Crocidura*	*Crocidura tanakae*	4	0 (0)	
	*Crocidura* sp.	1	0 (0)	
*Eothenomys*	*Eothenomys* sp.	21	0 (0)	
*Leopoldamys*	*Leopoldamys edwardsi*	89	6 (6.74)	4.48 (1.07–22.11)
*Microtus*	*Microtus fortis*	24	0 (0)	
*Mus*	*Mus musculus*	1	0 (0)	
	*Mus musculus castaneus*	2	0 (0)	
*Niviventer*	*Niviventer fulvescens*	2	0 (0)	
	*Niviventer lotipes*	98	22 (22.45)	14.93* (3.83–65.10)
*Rattus*	*Rattus nitidus*	24	0 (0)	
	*Rattus norvegicus*	434	9 (2.07)	1.38 (0.36–6.43)
	*Rattus rattus*	83	2 (2.41)	1.60 (0.25–10.36)
	*Rattus tanezumi*	133	2 (1.50)	1
*Suncus*	*Suncus murinus*	25	2 (8.00)	5.32 (0.79–34.63)
Unidentified		367	15 (4.09)	
Total		1,615	71 (4.40)	

OR: odd ratio, CI confidence interval; *p < 0.05, Chi-square Test.

### 3.5 Prevalence of *Babesia microti* in ticks

Among the 280 ticks collected, nine were found to be positive for *B. microti* (Table 5). These positive ticks were detected in Qingtian, Yunhe, and Jingning counties, but not in Tiantai. Although relatively few ticks were collected from the three surveillance sites, the infection rates were high. Notably, in Jingning, *B. microti* was detected in 2 of only 7 ticks collected. In contrast, Tiantai, where the largest number of ticks was collected (168 in 2023), yielded no *B. microti*-positive samples.

**Table 5:** Prevalence of *B. microti* in the ticks of different species.

Species	Tick source	No.	No. of positive (%)
*Haemaphysalis longicornis*	Questing	89 (TT = 89)	0 (0)
	Rodent-attached	79 (TT = 79)	0 (0)
*Ixodes granulatus*	Rodent-attached	5 (YH = 4, JN = 1)	5 (100.00)
*Rhipicephalus haemaphysaloides*	Rodent-attached	1 (QT = 1)	0 (0)
*Rhipicephalus microplus*	Rodent-attached	28 (QT = 28)	1 (3.57)
Unidentified	Rodent-attached	44 (JN = 6, QT = 2, YH = 36)	1 (2.27)
	Goat-attached	13 (QT = 13)	1 (7.70)
	Cattle-attached	21 (QT = 21)	1 (4.76)
Total		280	9 (3.21)

TT, Tiantai County; YH, Yunhe County; JN, Jingning County; QT, Qingtian County.

Of the 280 ticks collected, 202 were successfully identified to the species level (Table 5). The species composition and infection status are summarized in Table 5. Among these, *Haemaphysalis longicornis* (*n* = 168) and *Rhipicephalus haemaphysaloides* (*n* = 1) showed no detectable *B. microti* infections. In contrast, all five collected *Ixodes granulatus* ticks were positive for *B. microti*, and only one positive sample was detected among the 28 *Rhipicephalus microplus* ticks. No *B. microti* infection was detected in the questing ticks, whereas positive samples were observed in the host-attached ticks collected from rodents, goats, and cattle.

### 3.6 Sequencing and phylogenetic analysis of *Babesia microti*

Near-full-length 18S rRNA gene sequences were recovered from 28 of 86 *B. microti*-positive samples in this study, including 25 rodent-sourced, two tick-sourced, and one local resident-sourced samples. These sequences were deposited in GenBank with accession numbers PQ039648-PQ039675, and each sequence length ranges from 1,567 to 1736 bp.

**Figure 4:**
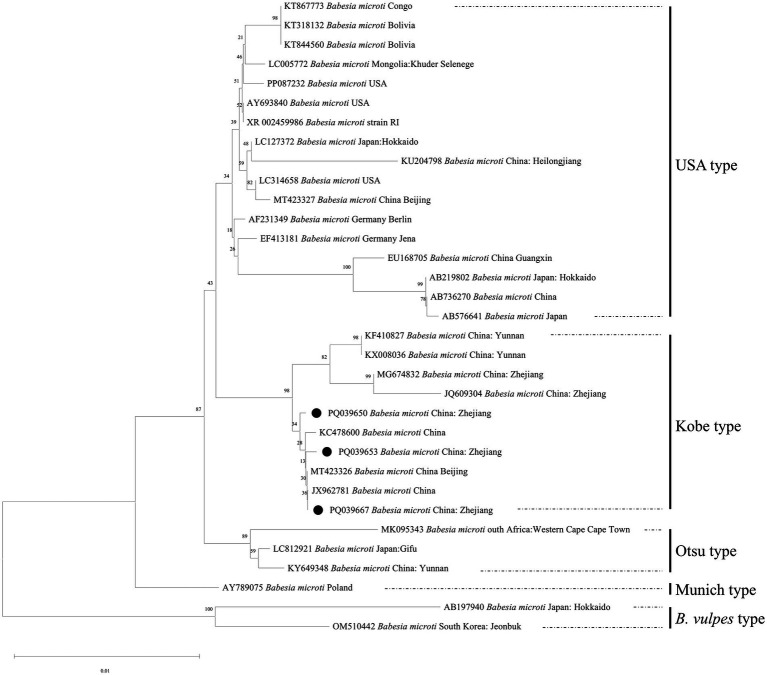
Neighbor-joining phylogenetic tree based on the comparison of *B. microti* 18S rRNA gene sequences obtained in this study with *B. microti* reference strains. The number on each branch denotes the percentage of occurrences in 1000 bootstrap replicates. Black squares stand (●) for sequences identified in this study. The branch lengths were drawn in proportion to the estimated sequence divergence.

Two unique sequences were obtained from a rat (PQ039650) and a local resident (PQ039653). The remaining sequences were identical, and one (PQ039667) was selected as a representative for inclusion in the phylogenetic analysis. The analysis revealed that these three haplotypes were closely related to samples (MG674832 and JQ609304) collected in other studies in the Zhejiang Province, and all belonged to *B. microti* Kobe type (Figure 4).

## 4 Discussion

This study presents a systematic, long-term investigation of *B. microti* throughout its transmission cycle. This included reservoir hosts, vector ticks, and human populations, and positive samples were obtained at every stage. This indicates that *B. microti* is widely distributed in the mountains of the southern Zhejiang Province and constitutes a latent reservoir for babesiosis.

**Figure 5:**
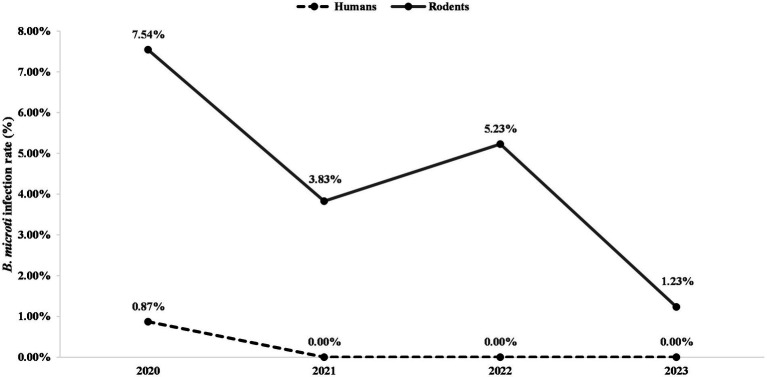
Annual infection rates of *B. microti* in humans and rodents in southern Zhejiang Province, 2020–2023.

In the present study, all of the human infections with *B. microti* were detected in 2020, which may be attributable to multiple factors. As observed in many zoonotic diseases, human infection risk has often been associated with infection levels in animal reservoirs (Keesing and Ostfeld, 2024, Plowright et al., 2017). In our study, the rodents captured in 2020 showed the highest *B. microti* infection rates during the surveillance period (Figure 5), which may have increased opportunities for spillover transmission to humans. In addition, vector distribution is an important determinant of pathogen transmission (Plowright et al., 2017). Previous surveillance in the Zhejiang Province reported higher tick densities in 2020 than in 2021 and 2022 (Wang et al., 2023), which may also have contributed to the increased number of human infections observed in that year.

The individuals infected with *B. microti* identified in this study were predominantly older farmers, which may be attributed to their lifestyles and the characteristics of the local agricultural environment. In the mountainous areas of southern Zhejiang Province, most cultivated fields are located along mountain forests, increasing farmers’ exposure to tick habitats, and consequently, increasing the risk of *B. microti* infection. Similarly, cases reported in the United States and England suggest that babesiosis in humans tends to occur more frequently in older age groups (Ho et al., 2021, Zabala et al., 2024). Data from a murine model of human babesiosis suggest that resistance to *B. microti* infection conferred by the adaptive immune system is genetically determined and altered by aging (Vannier et al., 2004). Age is a clear risk factor for severe *B. microti* infections in humans (Krause, 2002). Notably, all of the six infected individuals identified in this study were asymptomatic. In some cases, babesiosis may resolve without specific antimicrobial treatment in immunocompetent individuals (Vannier et al., 2015). The six infected individuals identified in this study may have been in an early asymptomatic phase of infection or in a stage of persistent low-level parasitemia following recovery, as parasitemia may persist for months to years after clinical recovery (Krause et al., 2003).

In 2018, the FDA released a draft guidance document (finalized in May 2019) mandating the testing of blood donations for *B. microti* infection in endemic US states (Administration, F. A. D, 2019). However, this test is not included in blood donor screening in China, even in areas where *Babesia* is endemic. One study was conducted with blood donors in Heilongjiang, China (Bloch et al., 2018). A total of 1,000 donor samples had been collected and evaluated using IFA against *B. microti*: 13/1000 (1.3%) were seroreactive. In Zhejiang, a case of death due to suspected transfusion-transmitted babesiosis (TTB) was reported (Xu et al., 2023), which undoubtedly represents a significant risk for TTB in China. This survey did not find any blood donors carrying *B. microti* but detected six positive blood samples from local residents, who are also potential blood donors. The prevalence in this population was found to be slightly higher than that reported by the American Red Cross (Moritz et al., 2016), thus suggesting a higher estimated risk in China without *Babesia* screening.

A history of tick bites is also a poor predictor of infection. Similar to the six *B. microti*–infected individuals identified in the present study, the recall of tick bites is unreliable. One study has observed no significant differences in *Babesia* seroprevalence between those who reported tick bites and those who did not (Leiby et al., 2002). Consequently, the prevention of TTB relies on the laboratory-based screening of blood donors (Bloch et al., 2021). Serological testing is relatively inexpensive and useful for assessing past exposure to *B. microti*, whereas qPCR allows direct detection of parasitemia and therefore reflects current infection status. These two approaches are complementary for understanding the epidemiology of babesiosis in the general population. In endemic regions, a combined screening strategy incorporating both serological assays and highly sensitive qPCR may further improve the detection of *Babesia* infections and help reduce the risk of transfusion-transmitted babesiosis.

As important reservoir hosts, rodents exhibit a high infection rate (4.40%). However, the prevalence of *B. microti* observed in this study differed noticeably from the previous survey results in Zhejiang. In the 2013 and 2017 surveys, the infection rates among rodents were 1.30 and 14.52%, respectively. These differences may be related to sampling site and time, as temperature and humidity significantly affect tick growth and reproduction (Gao et al., 2017). Notably, neighboring counties do not always have a similar prevalence of *B. microti*. For example, although Jingning is surrounded by three other counties in Lishui and close to Wencheng (Figure 1), it has the lowest rodent infection rate. This can be attributed to the different distributions and densities of rodents and ticks (Wei et al., 2020). As the least populated county in Lishui, Jingning has a lower rodent density than the surrounding counties (Luo et al., 2022), which may reduce the spread of *B. microti* among rodents to a certain extent. This suggests that local rodent density is an important risk factor for *B. microti*.

Among all rodent species, *N. lotipes* exhibited the highest risk of *B. microti* infection (positive rate = 22.45%, OR = 14.93), consistent with findings reported in Shanxi Province, China, where Liu *et al.* reported that *Niviventer confucianus* had the highest *B. microti* infection rate among local rodent populations (Liu et al., 2024). A survey on small mammals in Beijing yielded similar results (Wei et al., 2020). Although different *Niviventer* species predominate in the three regions, these studies highlight the role of the *Niviventer* genus as a significant reservoir host for *B. microti* in China. In addition, the higher inherent susceptibility of *Niviventer* genus to *B. microti* infection cannot be ruled out and warrants further investigation. Comparative experimental infection studies are valuable for confirming differences in host susceptibility among rodent species. In this survey, the common hosts of *B. microti* (*Apodemus*, *Rattus*, and *Suncus*) were captured, and positive individuals were identified for each genus. In contrast, *Peromyscus leucopus*, the primary reservoir of *B. microti* in the United States, is not trapped (Vannier et al., 2015). This contrast reflects substantial differences in the rodent host populations that transmit *B. microti* in different countries.

*Babesia microti* is now recognized as a genetically diverse species that includes several clades, including *B. microti* from the US, and *B. vulpes*, Munich, Kobe, and Otsu types (Liu et al., 2024). Previous studies have shown that different *B. microti* genotypes are associated with different tick vectors. The US type is primarily transmitted by *Ixodes scapularis* (Spielman, 1976), whereas the Munich and *B. vulpes* types are mainly associated with *Ixodes hexagonus* and *Ixodes trianguliceps*, respectively (Camacho et al., 2003, Bown et al., 2008). The Otsu type has been detected in *Ixodes ovatus*, and laboratory studies have confirmed its vector competence (Zamoto-Niikura et al., 2012). In contrast, the tick vector responsible for the transmission of the Kobe type remains largely undescribed (Goethert, 2021).

In the present study, Kobe type *B. microti* was detected in *I. granulatus* and *R. microplus*, with positive samples predominantly detected in *I. granulatus*. These findings suggest that these tick species may participate in the transmission cycle of the Kobe type in Zhejiang Province, with *I. granulatus* potentially playing an important local role. Notably, all 168 ticks collected from Tiantai were identified as *H. longicornis*, including 89 questing and 79 rodent-attached ticks, and no *B. microti* infections were detected (Table 5). This observation may reflect differences in vector associations among *B. microti*, which appear to be more frequently associated with ticks belonging to the *Ixodes* and *Rhipicephalus* genera rather than *Haemaphysalis*. Correspondingly, the variation in infection rates observed among sampling sites, as well as between questing and host-attached ticks, may also be influenced by such vector associations. The total infection rate of ticks was 3.21%, which is similar to the results of a survey conducted in Northeast China during 2018–2019 (Wang et al., 2020). As a tick-borne pathogen, the prevalence of *B. microti* in ticks showed inconsistencies in certain aspects, probably owing to sampling deviations. Ticks were collected only from Tiantai, Qingtian, Yunhe, and Jingning, and the sample sizes varied significantly. No *B. microti* were detected among the 168 ticks in Tiantai, probably because most of the collected ticks were questing rather than host-attached. Some studies have suggested that the zygotes of certain *Babesia* species can multiply and invade various tick organs, including the ovaries. This results in transovarial transmission for species such as *B. bovis* and *B. bigemina*, but not *B. microti.*

Different genotypes of *B. microti* show distinct geographical distribution patterns worldwide. The US type has been widely detected in North America, Europe, and Asia, although human babesiosis cases have been reported most frequently in the United States. The *B. vulpes* type is known to infect carnivores, including raccoons, foxes, and badgers worldwide, as well as sick domestic dogs in Spain. The Munich type has been primarily detected in voles and occurs in Europe and North America, but has not been reported in Asia. The Kobe and Otsu types have not been reported in Europe or North America, but are widely distributed in Japan and China (Goethert, 2021). In China, surveys have shown that isolates detected in the Heilongjiang and Shanxi provinces belong exclusively to the Kobe type (Sun et al., 2008, Ren et al., 2025), whereas both Kobe and Otsu types have been reported in Yunnan Province (Gao et al., 2017), and both the US and Kobe types have been identified in Beijing (Wei et al., 2020). All 28 sequences obtained in this study clustered into the Kobe type with those previously reported in Zhejiang Province, which is distinct from the US and Otsu types found in Beijing and Yunnan (Wei et al., 2020, Gao et al., 2017). The data show that the genotypes of *B. microti* varied according to the geographical region investigated.

## 5 Limitations

This study has some limitations: (1) Most younger individuals in the study areas migrated to urban areas for work, thus resulting in most participants being elderly residents. This demographic imbalance may distort the observed age distribution of the infections and limit the accuracy of age-related risk assessment. (2) Only a small number of ticks were captured, and the calculated *B. microti* infection rate in the ticks may be unreliable.

## 6 Conclusion

To the best of our knowledge, this is the first report of *B. microti* infection in three stages of its life cycle in Zhejiang Province. Our study revealed a wide prevalence of *B. microti* in rodents, ticks, and populations, all of which belonged to the Kobe type. Asymptomatic human infections have been identified, demonstrating an elevated risk of *B. microti* infection in older adults. The risk of TTB exists in the Zhejiang Province, highlighting the necessity of *Babesia* screening in blood donors. *N. lotipes* appears to be the predominant rodent reservoir host in Zhejiang Province. In addition, *I. granulatus* and *R. microplus* may serve as primary tick vectors for *B. microti* in this region. These results provide valuable information for developing preventive strategies against babesiosis in China.

## Data Availability

The datasets presented in this study can be found in online repositories. The names of the repository/repositories and accession number(s) can be found in the article/Supplementary material.
