# Supplementary material for: Molecular epidemiology of Babesia microti in southern Zhejiang: an integrated survey of humans, rodent reservoirs, and tick vectors (2020–2023)
Source: Front Microbiol. 2026 Mar 20;17:1799424. doi: 10.3389/fmicb.2026.1799424 (PMC13047210; doi:10.3389/fmicb.2026.1799424)
Supplement: Supplementary file 1 [file Table_1.docx]

**Additional file 1**

**Epidemiological Case Investigation Form for *Babesia* Infection**

1. Basic Patient Information

1.1 Patient name: ______________________________

1.2 Identification number: ______________________________

1.3 Contact telephone: ______________________________

1.4 Date of birth: ____ / ____ / ______ Age: ______ years

1.5 Sex: ① Male ② Female

1.6 Occupation: ______________________________

1.7 Registered residence (Province / City / County / Township): ______________________________

1.8 Current residence (Province / City / County / Township): ______________________________

1.9 GPS coordinates of current residence: ______________________________

2. Case Detection and Classification

2.1 Mode of case detection:

① Patient seeking medical care (e.g., fever patient blood examination, clinical visit)

② Active surveillance (monitoring samples, transfusion source tracing, etc.)

2.2 Presence of symptoms: ① Asymptomatic infection (skip to Section 4.1) ② Symptomatic

3. Onset of Illness and Medical Consultation

3.1 Main clinical manifestations (multiple choices allowed):

① Fever

② Chills

③ Sweating

④ Fatigue

⑤ Nausea

⑥ Loss of appetite

⑦ Myalgia

⑧ Arthralgia

⑨ Headache

⑩ Abdominal pain

⑪ Anemia or hemoglobinuria

⑫ Asymptomatic

⑬ Other: __________________

3.2 Presence of underlying diseases: ① Yes ② No (if no, skip to Section 3.4)

3.3 Underlying diseases: ______________________________

3.4 Date of onset: ____ / ____ / ______

3.5 Date of medical consultation: ____ / ____ / ______

3.6 Severity of illness: ① Mild (untreated or outpatient treatment) ② Severe (hospitalized) ③ Critical

3.7 Medical institution visited: ______________________________

3.8 Clinical diagnosis: ______________________________

3.9 Date of diagnosis: ____ / ____ / ______ Diagnostic institution: ______________________________

4. Laboratory Examination

4.1 Microscopy result for *Babesia*: ① Not performed ② Positive ③ Negative ④ Other __________

4.2 Molecular detection (*Babesia* nucleic acid test):

① Not performed ② Positive (species: __________) ③ Negative ④ Other __________

4.3 Date of testing: ____ / ____ / ______

4.4 Date of sample collection: ____ / ____ / ______

4.5 Testing institution: ______________________________

4.6 Complete blood count (CBC):

Date of test: ____ / ____ / ______

Platelet count: __________

Hemoglobin: __________

5. Treatment

5.1 Was drug treatment administered? ① Yes ② No (if no, skip following items)

5.2 Drug(s) used: ① Chloroquine phosphate ② Clindamycin ③ Other __________ ④ Unknown

5.3 Dosage: ______________________________

5.4 Hospitalization: ① Yes ② No

5.5 Date of first medication: ____ / ____ / ______

5.6 Date of last medication: ____ / ____ / ______

5.7 Treatment outcome:

① Recovered (*Babesia* no longer detected)

② Asymptomatic carrier (symptoms resolved but *Babesia* detectable)

③ Treatment ineffective

④ Death

6. Investigation of Infection Source

6.1 Outdoor activities (forest, farmland, grassland, parks, etc.): ① Yes ② No

6.2 Contact with animals:

① Rodents or other small mammals

② Pets (cats, dogs, etc.)

③ Livestock (cattle, sheep, pigs, etc.)

④ Other animals __________

⑤ No animal contact

6.3 Within two months before illness onset: ① Tick bite ② Blood transfusion ③ Organ transplantation

6.4 Suspected source of infection: ① Tick-borne transmission ② Blood transfusion or organ transplantation ③ Other __________

Investigation institution: __________________

Investigator: __________________

Date of investigation: ____ / ____ / ______
