# Supplementary material for: Molecular epidemiology of Babesia microti in southern Zhejiang: an integrated survey of humans, rodent reservoirs, and tick vectors (2020–2023)
Source: Front Microbiol. 2026 Mar 20;17:1799424. doi: 10.3389/fmicb.2026.1799424 (PMC13047210; doi:10.3389/fmicb.2026.1799424)
Supplement: Supplementary file 2 [file Table_2.docx]

| **Additional file 2. Primers and one probe used in this study to identify, amplify,** **and sequence the 18S rRNA gene of *B. microti* and to identify the rodent and tick species.** | | |
| --- | --- | --- |
| **Function** | **Name** | **Sequence (5' - 3')** |
| Identification of *B. microti* | Babesia-F | TCATTAAATTYGCTTCCGAGCG |
|  | Babesia-R | AAATCCCGGAAAATAGAACCCC |
|  | Babesia-PB | FAM-TGACTTGGCATCTTCTGGATTTGGTTCC-BHQ1 |
| Amplification of *B. microti* 18S rRNA gene | Prio2F | GCCAGTAGTCATATGCTTGTSTTA |
|  | Prio6R-ref | CTCCTTCCTTYAAGTGATAAGGTTCAC |
| Sequencing of *B. microti* 18S rRNA gene | Ba-SL | GGTTAATAGGAGCAGTTGGGGGCATTCG |
|  | Ba-SR | CTTTGATTTCTCTCAAGGTGCTGAAGGAGTC |
| Identification of rodent species | 16SarL | CGCCTGTTTAACAAAAACAT |
|  | 16SHm | AGATCACGTAGGACTTTAAT |
| Identification of tick species | 16S+1 | CTGCTCAATGATTTTTTAAATTGCTGTGG |
|  | 16S-1 | CCGGTCTGAACTCAGATCAAGT |
